# Bis(dimethyl sulfoxide-κ*O*)bis­(mercapto­acetato-κ^2^ *O*,*S*)tin(IV)

**DOI:** 10.1107/S160053680904361X

**Published:** 2009-10-28

**Authors:** Li Song

**Affiliations:** a Department of Chemistry, Key Laboratory of Advanced Textile Materials and Manufacturing Technology of the Ministry of Education, Zhejiang Sci-Tech University, Hangzhou 310018, People’s Republic of China

## Abstract

In the title compound, [Sn(C_2_H_2_O_2_S)_2_(C_2_H_6_OS)_2_], the mercaptoacetato ligands chelate to Sn^IV^ through S and one O atoms. The metal centre is also coordinated by two dimethyl sulfoxide (DMSO) ligands through the O atom, leading to an overall distorted octahedral coordination environment for the Sn^IV^ atom. The mol­ecular adduct lies on a twofold rotation axis.

## Related literature

For related structures of tin–mercaptoacetates, see: Holmes *et al.* (1988 ▶); Song *et al.* (1998 ▶); Ng *et al.* (1996 ▶); Zhang *et al.* (2006 ▶); Song *et al.* (2005 ▶); Wu *et al.* (2000 ▶); Zhong *et al.* (2004*a* ▶,*b* ▶, 2005*a* ▶,*b* ▶). For the chemistry of tin compounds, see: Smith (1998 ▶).
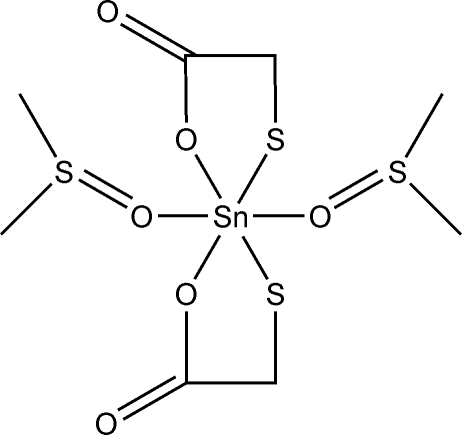


## Experimental

### 

#### Crystal data

[Sn(C_2_H_2_O_2_S)_2_(C_2_H_6_OS)_2_]
*M* *_r_* = 455.14
Monoclinic, 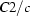

*a* = 13.3460 (17) Å
*b* = 8.2706 (7) Å
*c* = 14.9053 (18) Å
β = 107.124 (5)°
*V* = 1572.3 (3) Å^3^
*Z* = 4
Mo *K*α radiation
μ = 2.17 mm^−1^
*T* = 130 K
0.20 × 0.15 × 0.15 mm

#### Data collection

Rigaku R-AXIS RAPID diffractometer
Absorption correction: multi-scan (*ABSCOR*; Higashi, 1995 ▶) *T*_min_ = 0.671, *T*_max_ = 0.737
5801 measured reflections
1800 independent reflections
1718 reflections with *I* > 2σ(*I*)
*R*_int_ = 0.021

#### Refinement

*R*[*F* ^2^ > 2σ(*F* ^2^)] = 0.020
*wR*(*F* ^2^) = 0.047
*S* = 1.10
1800 reflections
87 parameters
H-atom parameters constrained
Δρ_max_ = 0.75 e Å^−3^
Δρ_min_ = −0.43 e Å^−3^

### 

Data collection: *PROCESS-AUTO* (Rigaku, 1998 ▶); cell refinement: *PROCESS-AUTO*; data reduction: *CrystalStructure* (Rigaku/MSC, 2004 ▶); program(s) used to solve structure: *SHELXS97* (Sheldrick, 2008 ▶); program(s) used to refine structure: *SHELXL97* (Sheldrick, 2008 ▶); molecular graphics: *SHELXTL* (Sheldrick, 2008 ▶); software used to prepare material for publication: *SHELXTL*.

## Supplementary Material

Crystal structure: contains datablocks I, global. DOI: 10.1107/S160053680904361X/ng2673sup1.cif

Structure factors: contains datablocks I. DOI: 10.1107/S160053680904361X/ng2673Isup2.hkl

Additional supplementary materials:  crystallographic information; 3D view; checkCIF report

## Figures and Tables

**Fig. 1.**
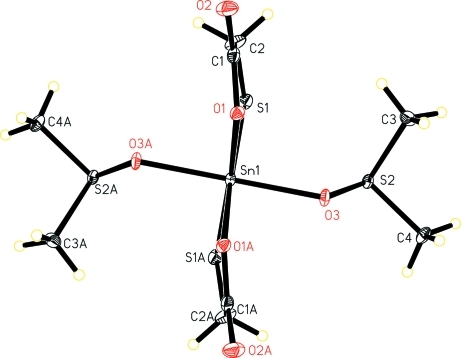
Structure and labeling of the title compound, with displacement ellipsoids drawn at the 30% probability level and H atoms shown as small spheres of arbitrary radii.

**Fig. 2.**
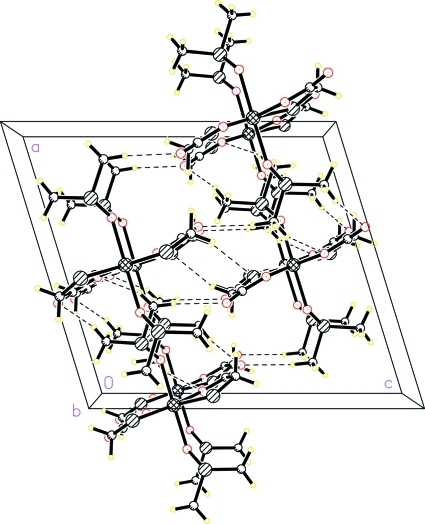
The packing diagram viewed along the b-direction.

## supplementary crystallographic information

### Comment

Compared with organotin compounds, inorganic compounds of tin are also important in industry applications, for example, electroplating, ceramic glazes and pigments,heterogeneous catalysts, gas sensors, and so on. (Smith *et al.*, 1998) Perhaps the most important recent development in tin (iv) chemistry has been the increase in studies of the solid state properties of tin (iv) compounds. Sn(SCH_2_CH_2_S)_2_ could act as a typical Lewis acid and reveal to be a electron acceptor. And many structures have been reported to exhibit the reaction of Sn(SCH_2_CH_2_S)_2_ and ligands. (Wu *et al.*, 2000; Holmes *et al.*, 1988) Here, the S-contained chelated ligand is mercapto acetic acid but not 1,2-ethanedithiol ligand, and the solvent DMSO act as the second ligand.

The title compound, Sn(C_2_H_2_O_2_S)_2_(DMSO)_2_, is a mononuclear structure and crystallizes in monoclinic form in the space group *C*2/*c*. As shown in Figure 1, the asymmetric unit is composed of half tin atom, one mercaptoacetato and one DMSO ligand. According to a *C*2 symmetry axis pass the tin (iv) site, a mononuclear structure is present. In which, two mercaptoacetato ligands coordinates to Sn^IV^ through S and one O atoms. The metal centre is also coordinated by two dimethyl sulfoxide ligands through O atom, froming a SnO_4_S_2_ distorted octahedronal coordianted sphere. Around the metal centre, two mercaptoacetato ligands adopt *cis* chelated mode to form a SnO_2_S_2_ distorted equatorial plan. And other two DMSO ligands join on it from two polars of the coordinated sphere, also with *cis* mode around the metal centre.

### Experimental

All chemicals were obtained from commercial sources and were used as received. The title compound was handily synthesized by a solution reaction from mercapto acetic acid. HSCH_2_COOH (56 mg, 0.6 mmol) and NaOH (50 mg, 1.2 mmol) was dissolved in 10 ml of water. To this solution was added a 5 ml aqueous solution of SnCl_4_.5H_2_O (106 mg, 0.3 mmol) at room temperature. Amount of white precipitates were gradually formed and colected by filtrating and washing with water. Then they were dissolved in 5 ml DMSO and the filtration was slowly evaperated at room temperature. After several days, a great deal of colorless crystals were obtained, yield about 113 mg (83% on tin).

### Refinement

The structure was solved using direct methods and refined by full-matrix least-squares techniques. All non-hydrogen atoms were assigned anisotropic displacement parameters in the refinement. All hydrogen atoms were added at calculated positions and refined using a riding model. The structure was refined on F2 using *SHELXTL97* software package(Sheldrick *et al.*, 2008) without any unusual events.

### Crystal data

**Table:**

[Sn(C_2_H_2_O_2_S)_2_(C_2_H_6_OS)_2_]	*F*(000) = 904
*M**_r_* = 455.14	*D*_x_ = 1.923 Mg m^−^^3^
Monoclinic, *C*2/*c*	Mo *K*α radiation, λ = 0.71075 Å
Hall symbol: -C 2yc	Cell parameters from 2229 reflections
*a* = 13.3460 (17) Å	θ = 3.1–27.5°
*b* = 8.2706 (7) Å	µ = 2.17 mm^−^^1^
*c* = 14.9053 (18) Å	*T* = 130 K
β = 107.124 (5)°	Prism, white
*V* = 1572.3 (3) Å^3^	0.20 × 0.15 × 0.15 mm
*Z* = 4	

### Data collection

**Table:**

Rigaku R-AXIS RAPID diffractometer	1800 independent reflections
Radiation source: fine-focus sealed tube	1718 reflections with *I* > 2σ(*I*)
graphite	*R*_int_ = 0.021
Detector resolution: 14.6306 pixels mm^-1^	θ_max_ = 27.5°, θ_min_ = 3.2°
CCD_Profile_fitting scans	*h* = −11→17
Absorption correction: multi-scan (*ABSCOR*; Higashi, 1995)	*k* = −10→10
*T*_min_ = 0.671, *T*_max_ = 0.737	*l* = −19→19
5801 measured reflections	

### Refinement

**Table:**

Refinement on *F*^2^	Primary atom site location: structure-invariant direct methods
Least-squares matrix: full	Secondary atom site location: difference Fourier map
*R*[*F*^2^ > 2σ(*F*^2^)] = 0.020	Hydrogen site location: inferred from neighbouring sites
*wR*(*F*^2^) = 0.047	H-atom parameters constrained
*S* = 1.10	*w* = 1/[σ^2^(*F*_o_^2^) + (0.0207*P*)^2^ + 2.5592*P*] where *P* = (*F*_o_^2^ + 2*F*_c_^2^)/3
1800 reflections	(Δ/σ)_max_ = 0.001
87 parameters	Δρ_max_ = 0.75 e Å^−^^3^
0 restraints	Δρ_min_ = −0.43 e Å^−^^3^

### Special details

**Table:**

Geometry. All e.s.d.'s (except the e.s.d. in the dihedral angle between two l.s. planes) are estimated using the full covariance matrix. The cell e.s.d.'s are taken into account individually in the estimation of e.s.d.'s in distances, angles and torsion angles; correlations between e.s.d.'s in cell parameters are only used when they are defined by crystal symmetry. An approximate (isotropic) treatment of cell e.s.d.'s is used for estimating e.s.d.'s involving l.s. planes.
Refinement. Refinement of *F*^2^ against ALL reflections. The weighted *R*-factor *wR* and goodness of fit *S* are based on *F*^2^, conventional *R*-factors *R* are based on *F*, with *F* set to zero for negative *F*^2^. The threshold expression of *F*^2^ > σ(*F*^2^) is used only for calculating *R*-factors(gt) *etc*. and is not relevant to the choice of reflections for refinement. *R*-factors based on *F*^2^ are statistically about twice as large as those based on *F*, and *R*- factors based on ALL data will be even larger.

### Fractional atomic coordinates and isotropic or equivalent isotropic displacement parameters (Å^2^)

**Table:**

	*x*	*y*	*z*	*U*_iso_*/*U*_eq_	
Sn1	0.0000	0.20692 (2)	0.7500	0.01316 (7)	
S1	−0.04306 (4)	0.02254 (7)	0.61815 (4)	0.02162 (12)	
S2	0.22228 (4)	0.12036 (6)	0.71261 (3)	0.01444 (11)	
O1	−0.04086 (11)	0.39054 (17)	0.65158 (10)	0.0164 (3)	
O2	−0.13288 (14)	0.4586 (2)	0.50766 (10)	0.0285 (4)	
O3	0.16014 (11)	0.24764 (18)	0.75112 (10)	0.0179 (3)	
C1	−0.09221 (16)	0.3555 (3)	0.56564 (14)	0.0194 (4)	
C2	−0.1050 (2)	0.1784 (3)	0.53429 (16)	0.0296 (5)	
H2B	−0.0781	0.1677	0.4794	0.036*	
H2A	−0.1811	0.1547	0.5122	0.036*	
C3	0.22763 (19)	0.2033 (3)	0.60389 (15)	0.0239 (5)	
H3A	0.1588	0.1928	0.5571	0.036*	
H3B	0.2801	0.1449	0.5823	0.036*	
H3C	0.2469	0.3178	0.6124	0.036*	
C4	0.35322 (16)	0.1546 (3)	0.78190 (16)	0.0215 (4)	
H4A	0.3626	0.1143	0.8457	0.032*	
H4B	0.3682	0.2708	0.7842	0.032*	
H4C	0.4013	0.0978	0.7542	0.032*	

### Atomic displacement parameters (Å^2^)

**Table:**

	*U*^11^	*U*^22^	*U*^33^	*U*^12^	*U*^13^	*U*^23^
Sn1	0.00996 (10)	0.01364 (11)	0.01662 (10)	0.000	0.00506 (7)	0.000
S1	0.0191 (3)	0.0178 (3)	0.0260 (3)	0.0000 (2)	0.0035 (2)	−0.0068 (2)
S2	0.0111 (2)	0.0132 (2)	0.0194 (2)	0.00096 (18)	0.00510 (19)	−0.00037 (17)
O1	0.0143 (7)	0.0163 (7)	0.0177 (6)	−0.0018 (6)	0.0033 (6)	0.0011 (5)
O2	0.0319 (9)	0.0303 (9)	0.0192 (7)	0.0098 (7)	0.0013 (7)	0.0025 (7)
O3	0.0106 (7)	0.0193 (7)	0.0259 (7)	−0.0017 (6)	0.0086 (6)	−0.0053 (6)
C1	0.0132 (10)	0.0253 (11)	0.0206 (10)	0.0041 (8)	0.0063 (8)	−0.0018 (8)
C2	0.0264 (12)	0.0304 (13)	0.0231 (11)	0.0113 (10)	−0.0065 (10)	−0.0076 (9)
C3	0.0250 (12)	0.0303 (12)	0.0180 (9)	0.0026 (9)	0.0088 (9)	0.0008 (9)
C4	0.0117 (10)	0.0217 (10)	0.0280 (11)	0.0037 (8)	0.0013 (9)	−0.0023 (9)

### Geometric parameters (Å, °)

**Table:**

Sn1—O1^i^	2.0699 (14)	O2—C1	1.222 (3)
Sn1—O1	2.0699 (14)	C1—C2	1.531 (3)
Sn1—O3	2.1587 (14)	C2—H2B	0.9900
Sn1—O3^i^	2.1587 (14)	C2—H2A	0.9900
Sn1—S1	2.4193 (6)	C3—H3A	0.9800
Sn1—S1^i^	2.4193 (6)	C3—H3B	0.9800
S1—C2	1.817 (2)	C3—H3C	0.9800
S2—O3	1.5511 (15)	C4—H4A	0.9800
S2—C4	1.771 (2)	C4—H4B	0.9800
S2—C3	1.780 (2)	C4—H4C	0.9800
O1—C1	1.295 (2)		
			
O1^i^—Sn1—O1	85.61 (8)	O2—C1—O1	122.6 (2)
O1^i^—Sn1—O3	80.01 (6)	O2—C1—C2	117.72 (19)
O1—Sn1—O3	86.83 (6)	O1—C1—C2	119.67 (19)
O1^i^—Sn1—O3^i^	86.83 (6)	C1—C2—S1	118.77 (16)
O1—Sn1—O3^i^	80.01 (6)	C1—C2—H2B	107.6
O3—Sn1—O3^i^	162.05 (8)	S1—C2—H2B	107.6
O1^i^—Sn1—S1	171.18 (4)	C1—C2—H2A	107.6
O1—Sn1—S1	86.38 (4)	S1—C2—H2A	107.6
O3—Sn1—S1	95.88 (4)	H2B—C2—H2A	107.1
O3^i^—Sn1—S1	95.41 (4)	S2—C3—H3A	109.5
O1^i^—Sn1—S1^i^	86.38 (4)	S2—C3—H3B	109.5
O1—Sn1—S1^i^	171.18 (4)	H3A—C3—H3B	109.5
O3—Sn1—S1^i^	95.41 (4)	S2—C3—H3C	109.5
O3^i^—Sn1—S1^i^	95.88 (4)	H3A—C3—H3C	109.5
S1—Sn1—S1^i^	101.85 (3)	H3B—C3—H3C	109.5
C2—S1—Sn1	93.60 (8)	S2—C4—H4A	109.5
O3—S2—C4	102.64 (9)	S2—C4—H4B	109.5
O3—S2—C3	104.15 (10)	H4A—C4—H4B	109.5
C4—S2—C3	99.90 (11)	S2—C4—H4C	109.5
C1—O1—Sn1	119.26 (14)	H4A—C4—H4C	109.5
S2—O3—Sn1	121.82 (8)	H4B—C4—H4C	109.5
			
O1^i^—Sn1—S1—C2	−36.3 (3)	C3—S2—O3—Sn1	106.14 (12)
O1—Sn1—S1—C2	−11.53 (10)	O1^i^—Sn1—O3—S2	161.54 (11)
O3—Sn1—S1—C2	−97.96 (10)	O1—Sn1—O3—S2	−112.37 (10)
O3^i^—Sn1—S1—C2	68.06 (10)	O3^i^—Sn1—O3—S2	−155.05 (10)
S1^i^—Sn1—S1—C2	165.24 (9)	S1—Sn1—O3—S2	−26.35 (10)
O1^i^—Sn1—O1—C1	−169.37 (17)	S1^i^—Sn1—O3—S2	76.19 (10)
O3—Sn1—O1—C1	110.43 (15)	Sn1—O1—C1—O2	168.26 (17)
O3^i^—Sn1—O1—C1	−81.82 (15)	Sn1—O1—C1—C2	−10.7 (3)
S1—Sn1—O1—C1	14.32 (14)	O2—C1—C2—S1	178.66 (18)
S1^i^—Sn1—O1—C1	−144.6 (2)	O1—C1—C2—S1	−2.3 (3)
C4—S2—O3—Sn1	−150.07 (11)	Sn1—S1—C2—C1	10.8 (2)

Symmetry codes: (i) −*x*, *y*, −*z*+3/2.
